# Closing in on the causes of chronic neuropathic pain

**DOI:** 10.7554/eLife.111218

**Published:** 2026-03-18

**Authors:** Geoffroy Laumet

**Affiliations:** 1 Department of Physiology, Michigan State University East Lansing United States

**Keywords:** neuropathic pain, mRNA translation, spinal cord, dorsal root ganglia, gene expression, inhibitory neurons, Mouse

## Abstract

Chronic neuropathic pain in mice is maintained by the translation of messenger RNA in inhibitory neurons, so drugs that modulate translational pathways may be able to treat this condition.

**Related research article** Lister KC, Wong C, Uttam S, Parisien M, Stecum P, Brown N, Cai W, Hooshmandi M, Gu N, Amiri M, Beaudry F, Jafarnejad SM, Tavares-Ferreira D, Inturi NN, Mazhar K, Zhao HT, Fitzsimmons B, Gkogkas CG, Sonenberg N, Price TJ, Diatchenko L, Atlasi Y, Mogil JS, Khoutorsky A. 2024. Translational control in the spinal cord regulates gene expression and pain hypersensitivity in the chronic phase of neuropathic pain. *eLife* **13**:RP100451. doi: 10.7554/eLife.100451.

Neuropathic pain develops when nerve damage causes the pain-sensing pathways in the nervous system to become hypersensitive. It can persist for months or years, and symptoms include extreme sensitivity to touch and experiencing pain for no obvious reason. Current treatments include opioids and non-steroidal anti-inflammatory drugs (such as ibuprofen and aspirin), but these often provide only limited relief, underscoring the need to identify new therapeutic targets (Finnerup et al., 2015).

To date, research into neuropathic pain has tended to focus on the early (acute) stages of the condition, with less emphasis on the later (chronic) stages (LaCroix-Fralish et al., 2011). During the acute phase, neuropathic pain is driven by both transcriptional and translational regulation in neurons in the spinal cord and the dorsal root ganglia (which are attached to the spinal cord). However, the relative importance of transcription (the process by which DNA makes messenger RNA) and translation (the process by which ribosomes read messenger RNA to build proteins) in the chronic phase has been unclear (Ray et al., 2023).

Now, in eLife, Arkady Khoutorsky, Jeffrey Mogil and colleagues – including Kevin Lister of McGill University as first author – report the results of experiments on a mouse model of neuropathic pain in which they probe both transcription and translation during the acute (day 4) and chronic (~2 months) phases of the condition (Lister et al., 2024). Unexpectedly, they discovered that gene expression in the spinal cord during the chronic phase is governed by translation rather than transcription.

To test whether altered protein synthesis in the spinal cord contributes to chronic pain, the researchers inhibited eIF4E, a protein that is involving in initiating the process of translation (Gingras et al., 2001). They found that blocking translation in the spinal cord was sufficient to alleviate chronic neuropathic pain in mice, without causing detectable motor or cognitive impairments.

Lister et al. then used a visualization technique called FUNCAT (short for fluorescent non-canonical amino acid tagging) to see if the proteins involved in neuropathic pain were being synthesized in excitatory neurons, which transmit pain signals to the brain, or inhibitory neurons, which dampen pain signaling. Strikingly, most of the increase in protein synthesis occurred in inhibitory neurons.

To strengthen this observation, the researchers turned to a technique called TRAP (short for translating ribosome affinity purification) that can isolate ribosomes that are in the process of translating messenger RNA into proteins. Lister et al. used TRAP to study a subset of excitatory neurons (defined by a gene called *Tac1*) and a subset of inhibitory neurons (defined by a gene called *Gad2*). This analysis confirmed that most of the translational changes occurring in the spinal cord during the chronic phase of neuropathic pain take place in inhibitory neurons.

Next, to see if these translational changes were sufficient to drive chronic pain, the researchers disabled a protein that inhibits translation by binding to eIF4E. This manipulation was enough to induce persistent pain and reduce the electrical activity of two types of inhibitory neurons. In contrast, performing the same manipulation in excitatory neurons had no detectable effect on pain.

Finally, Lister et al. selectively blocked translation in a specific subtype of inhibitory neurons that is known to regulate mechanical pain. (They did this by using a short hairpin RNA molecule to target eIF4E, which is involved in starting the process of translation). This intervention restored the normal electrical activity of these neurons but, surprisingly, did not alleviate pain behavior. This finding suggests that inhibiting translation in a single subtype of inhibitory neuron is not enough on its own to reduce neuropathic pain, and that a broader range of inhibitory neurons has to be targeted to ensure effective pain relief.

Together, the work of Lister et al. – who are based at McGill and other institutions in Canada, the United States, the United Kingdom and Greece – represents a technical tour de force and provides the first evidence that increased translation in inhibitory neurons in the spinal cord is a key mechanism for maintaining chronic neuropathic pain in mice. An important next step will be to determine whether similar mechanisms occur in the spinal cords of human with neuropathic pain. If confirmed, targeting protein translation could offer new therapeutic strategies for individuals whose chronic pain is resistant to current treatments. Notably, several drugs that modulate translational pathways are already under development (Uttam et al., 2018). Inhibiting protein synthesis in the spinal cord may therefore represent a paradigm shift in the treatment of chronic neuropathic pain.
